# Survey of caregivers in Kenya to assess perceptions of zinc as a treatment for diarrhea in young children and adherence to recommended treatment behaviors

**DOI:** 10.7189/jogh.03.010405

**Published:** 2013-06

**Authors:** Evan Simpson, Greg Zwisler, Melissa Moodley

**Affiliations:** 1 PATH, Seattle, WA, USA; 2 IPSOS Healthcare, London, UK

## Abstract

**Background:**

In 2004, the United Nations Children’s Fund (UNICEF) and the World Health Organization (WHO) revised their recommendations for management of acute diarrhea in children to include zinc treatment as well as oral rehydration solution (ORS). Little is known about how caregivers in low–resource settings perceive and use zinc treatment.

**Methods:**

Using a semi–structured quantitative survey, we interviewed Kenyan caregivers who had used zinc to treat children aged 6–60 months with an episode of diarrhea during the previous 6 months. The survey asked about experience using zinc, compliance with course and dosing regimens, and the attributes of zinc compared with other treatments. We surveyed a quota sample of 100 women from several communities where zinc treatment was available, primarily through public sector providers.

**Results:**

The mean duration of the reference diarrhea episode was 5.3 days (95% confidence interval (CI) 4.7–5.9). Eighty–two respondents had used zinc tablets, and 18 had given zinc syrup. Among those who used tablets, 62% reported giving zinc for fewer than the recommended 10 days, with a mean of 6.8 days (95% CI 6.1–7.4 days), and 50% said they had been instructed to give zinc for 5 days or less. Also, only 55% gave the correct daily dose. When asked about other treatments, 64% of the respondents reported using antibiotics, 59% ORS, and 56% a homemade remedy. Among the zinc tablet users, 55% provided zinc as the 3^rd^ or 4^th^ treatment for the reference episode. Also, 75% of respondents reported receiving the zinc treatment free of charge. Caregivers reported a very high level of satisfaction with zinc treatment, with 88% indicating that zinc (either in tablet or syrup form) was their most preferred treatment.

**Conclusions:**

Despite the potential benefits of zinc for children with acute diarrhea in low–resource settings, treatment regimens remain unwieldy and unrealistic, perhaps unnecessarily. Furthermore, the availability of zinc is limited primarily to public–sector providers. Increasing access to this treatment beyond the public clinic or hospital may accelerate uptake and sustained use.

Globally, diarrhea remains one of the most significant causes of under–five mortality. It leads to an estimated 800 000 deaths annually in this age group, predominantly in developing countries [1]. In 2004, the World Health Organization (WHO) revised its recommendations for the management of acute diarrhea to include zinc treatment in conjunction with the administration of oral rehydration solution (ORS). Specifically, these guidelines recommend 20 mg of zinc per day at the onset of illness for 10 to 14 days (10 mg for infants under 6 months of age) [2]. These recommendations are based on evidence indicating that zinc can reduce the duration and severity of diarrhea and help to prevent recurrence for up to2 to 3 months [3]. In addition, some studies suggest that the promotion and use of zinc as a diarrhea treatment can reduce the inappropriate use of antibiotics [4].

Previous studies have indicated that caregivers sometimes use more than one intervention for treating diarrhea, and zinc has joined a number of interventions available to caregivers [5]. As with other interventions, zinc is not always used in isolation or just with ORS. Instead, it may be used in conjunction with one or more additional interventions. Some of these interventions, such as antibiotics and antimotility drugs, are not recommended for use in children in most cases. Others, such as home remedies, are valid components of oral rehydration therapy.

Because of the relatively recent addition of zinc to the portfolio of products for treating diarrhea in children, and the slow pace of uptake, there is limited information outside of controlled research settings on the behaviors of caregivers who have provided zinc to children, their ability to follow the therapy regimen, and their treatment preferences. Information from caregivers about how well zinc performs relative to other products, and their perceptions of the competing products, would be useful in developing programs aimed at increasing zinc use.

This article reports several key findings from a recent survey of caregivers in a rural area of Kenya about their behaviors and experience with using zinc to treat diarrhea in young children, their treatment and dosing practices, and views on how zinc compares with other interventions.

## Methods

### Participants

We surveyed a quota sample of 100 caregivers in Kenya’s Bungoma district. Interviewers visited households and administered a recruitment questionnaire to determine if they met the criteria for inclusion in the study. Key inclusion criteria included recent experience of diarrhea (within last six months), awareness of zinc and use of zinc in treating diarrhea in a child between 6 and 60 months old. We used a semi–structured oral survey with both open–ended and close–ended questions and interviewed caregivers in their homes. Because of the low coverage of zinc treatment in Kenya (as in most countries), survey participants were recruited from households with children in communities in Bungoma where this treatment was generally available through public or private health providers, clinics, and hospitals.

Participants were recruited in several villages (Sangalo, Kulisiru, Naitiri, Bungoma town, Kituni) within Bungoma district in Kenya’s Western Province, a predominantly agricultural area. In these communities, blister packs of ten 20 mg tablets were generally available through public–sector clinics and hospitals as part of a pilot effort to introduce zinc for the treatment of diarrhea. Providers in these areas had received some training about new guidelines for diarrhea management that include use of zinc. In addition, a modest radio campaign in these areas had included information on both prevention of diarrhea (hand washing, sanitation, etc.) and treatment using ORS and zinc. Both the training and the media campaign had concluded approximately 18 months before the survey was conducted.

### Survey

An original survey was created for the purpose of this study. An initial draft was pilot tested for appropriateness and effectiveness, and to determine the usefulness and completeness of the responses. Based on the results of the pilot test the survey instrument was finalized. The implementation of the survey took approximately one hour to complete with most of the mothers.

The survey asked specifically about the most recent case of diarrhea in which zinc was administered. Survey questions elicited information pertaining to treatment practices, caregivers’ overall satisfaction with use of zinc, comparisons with other products used, and understanding of the appropriate dosing regimen. To assist with recall and to overcome a respondent’s potential inability to identify brand names of medicines or classifications, several areas of questioning relied on the use of pictures of locally available products (antibiotics, ORS, etc.). In all cases, the surveys were given in local languages.

### Data analysis and presentation

In the case of the categorical questions, frequencies have been reported. In the case of numeric questions, the full distributions of results, including mean, as well as median have been reported. Median has been reported where relevant in consideration of the sample and type of questions being analyzed. As the survey was designed to provide a snapshot of attitudes and behaviors within a relatively small sample, the emphasis was on identifying the typical patterns that emerge. Based on this, the median provides a good measure of typical behaviors in terms of actual number of days of episode or number of tablets given as opposed to the mean which provides the statistical average but does not necessarily represent a typical behaviour.

The data were entered into computer and then analyzed using SPSS. Throughout the study, the market research agency (IPSOS Healthcare) maintained its standing permissions from the relevant authority in Kenya to conduct market and social research such as this study. IPSOS also adheres to the ICC/ESOMAR Code on the ethical conduct of market and social research [6]. This study was determined to be non–research by PATH’s Research Ethics Committee. Individuals’ participation in the surveys was entirely voluntary and anonymous, and all data was aggregated.

## Results

### Respondent demographics

All surveyed caregivers were women who had used zinc to treat diarrhea in a child between 6 and 60 months within the past 6 months. **Table 1** summarizes the demographics of the participants. Approximately two–thirds were less than 30 years old. More than a half were working full or part time. Most came from the two lowest income groups, which is consistent with the rural, low–income status of the region.

**Table 1:** Percentage distribution of respondents by demographic characteristics

Characteristic	% of respondents (n = 100)
Age:	
18–24 y	37
25–29	25
30–34	16
35 or more	22
Employment status:	
Not working	41
Working full time	22
Working part time	29
Unemployed, looking for work	8
Socio–economic grade:*	
C1 – Supervisory, clerical and junior managerial, administrative or professional	13
C2 – Skilled manual workers	42
D – Semi and unskilled manual workers	45

*In Kenya, the socio–economic grades as initially defined by the British National Readership Survey (and often used in market research globally) were used with adapted criteria in common use among members of the Marketing and Social Research Association of Kenya [7].

### Duration of illness and treatments used

The mean duration of illness for which the caretaker provided zinc was 5.3 days (95% confidence interval (CI) 4.7–5.9), with approximately one–third of the cases lasting 6 days or more (**Table 2**). Ninety caregivers reported using at least two treatments, and 30 reported using at least four treatments in the reference episode (**Table 3**). Sixty–four reported using antibiotics for the same bout of illness, 59 used ORS sachets, and 56 used a home remedy. Forty–nine gave a home remedy as their first treatment. Among the 82 who administered zinc tablets, 19% administered the product as the 3^rd^ treatment and 26% as the 4^th^ treatment.

**Table 2:** Duration of reference diarrhea episode (zinc was typically started on day 3)

No. of days	% of respondents (n = 100)
1	5
2	5
3	11
4	25
5	20
6–10	28
More than 10	6
Median	5
Mean	5.3 (95% CI 4.7–5.9)

CI – confidence interval

**Table 3:** Number and sequence of treatments used in the reference episode of diarrhea (n = 100 respondents)*

Treatment type	All treatments in reference episode†	First treatment used	Second treatment used	Third treatment used	Fourth treatment used
Homemade remedy	56	49	4	–	1
Powdered ORS	59	13	26	8	–
Zinc tablets	82	7	29	19	26
Zinc syrup	18	5	7	4	2
Antibiotics	64	26	20	16	1
Antimotility	13	–	4	6	–

*Questions: When your child had this diarrhea episode, did you give anything shown on this illustration apart from anything you have already mentioned in relation to foods or diet? What did you use first? What did you use second? Third? Fourth?

†Some respondents indicated using more than four treatments. As a result, the sum of four treatments used may not total all treatments used.

### Therapy regimen and dosing

**Figure 1:**
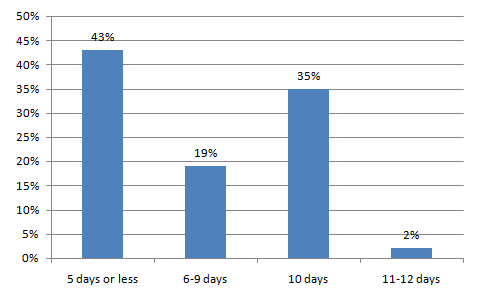
Duration of zinc therapy in days (n = 82 respondents).

The respondents were asked a series of questions about the zinc therapy and dosage regimen they had followed. Out of 82 zinc tablet users, 62% indicated that they had administered zinc for fewer than the recommended 10 days (mean = 6.8 days), 35% reported giving zinc for 10 days, and 2% administered zinc for 11 or 12 days (**Figure 1**). Among those who had given zinc for fewer than 10 days, 4 in 10 reported that they had received instructions to administer zinc for 10 days but did not do so, and approximately 5 in 10 reported they had been instructed to provide zinc for 5 days or less.

**Figure 2:**
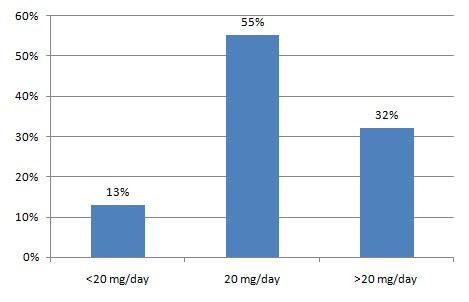
Daily dosage of zinc tablets (n = 82 respondents).

Among the zinc tablet users, 13% reported giving less than the recommended daily dose (20 mg per day), 55% gave the correct daily dose, and 32% gave more than the recommended dose (**Figure 2**). Typically, those who under–dosed gave half a tablet per day and those who over–dosed gave one tablet twice per day.

### Source and cost

Among those who used zinc tablets, 92% reported accessing the product through a public source, either a government hospital (60%) or a government health center/clinic (32%) (**Table 4**). About 6% reported receiving the product from a private clinic or provider. In all instances, the number of tablets provided was 10, which is the total number of tablets in a blister pack typically available in Kenya. Among 18 respondents who used zinc syrup, most received the syrup from a public source. Only 9% of tablet users and 17% of syrup users reported paying for the product. Also, 75 respondents reported receiving ORS when they received the zinc.

**Table 4:** Source of zinc and median number of tablets provided

Source	% of respondents using zinc tablets (n = 82)	Median number of zinc tablets provided	Mean number of zinc tablets provided (95% CI)	% of respondents using zinc syrup (n = 18)
Government health center or clinic	32	10	7.98 (7.01–8.93)	22
Government hospital	60	10	8.3 (7.56–9.04)	61
Private pharmacy	1	10	10	6
Private clinic	5	10	10	6
Other	2	n/a		6

CI – confidence interval

### Zinc treatment experience

Ninety–one women indicated that the zinc caused no negative effects on the child. Four, however, reported nausea, 3 reported dizziness, and 2 could not recall whether there were side effects. Despite minor, negative side effects in a few children, all 100 respondents said they would use zinc again the next time their child had diarrhea.

### Perceptions of zinc attributes

Most respondents had previously used other diarrhea treatments, with the most common being antibiotics, ORS, and home remedies (**Table 5**). Seventy respondents indicated that zinc tablets were their most preferred product, and 18 expressed a preference for zinc syrups. Although 91 respondents were aware of antibiotics and 82 had previously used them, only 5 indicated that antibiotics were their most preferred treatment.

**Table 5:** Caregivers’ awareness of and experience with various diarrhea treatments and their most preferred treatment (n = 100 respondents)

Treatment	Aware of the treatment (%)	Ever used the treatment (%)	Most preferred treatment (%)
Antibiotics	91	82	5
ORS	85	77	6
Zinc tablets	83	82	70
Home remedy	95	61	0
Antimotility drugs	49	19	1
Zinc syrups	28	22	18

ORS – oral rehydration solution

Respondents who preferred zinc tablets over other products gave a number of reasons for this preference. About 74% reported that it “stopped the diarrhea,” 60% said it “worked faster,” and 40% said it “restores energy” (**Table 6**).

**Table 6:** Reasons for preferring zinc over other products

Reason cited for preferring zinc	% respondents preferring zinc tablets (n = 70)	% respondents preferring zinc syrups (n = 18)
Effective – it stops diarrhea	74	61
It works faster	60	50
Restores energy	40	39
It helps the child gain appetite	20	17
Easily prepared/administered	13	11
It’s cheap	6	6
Recommended by doctors	6	11
It’s free of charge	6	11

When asked what they believed zinc would do for their child based on their experience, 82% of caregivers first mentioned “stop the diarrhea” (**Table 7**). Attributes typically mentioned second were “improve the appearance of stools” (20%), “Help treat the diarrhea faster, so diarrhea did not last as long” (18%), and“ reduce the frequency of bowel movements” (17%). Other commonly noted expectations included “improve child’s energy level”, “improve the child’s appetite”, and “give protection against future episodes of diarrhea.”

**Table 7:** Caregivers’ expectations for zinc treatment based on experience (n = 100 respondents)

Expectation about what zinc would do for child	1^st^ choice (%)	2^nd^ choice (%)	3^rd^ choice (%)	4^th^ choice (%)	5^th^ choice (%)
Stop the diarrhea	82	9	1	1	0
Reduce frequency of bowel movements	9	17	7	1	0
Help treat the diarrhea faster, so diarrhea did not last as long	5	18	8	2	0
Improve appearance of stools	2	20	5	1	0
Improve child’s energy level	0	8	10	3	1
Improve child’s appetite	0	10	13	4	1
Improve child’s health	0	3	5	6	2
Improve look of child’s eyes	0	1	1	–	1
Give protection against future episodes of diarrhea	0	2	2	2	0
Reduce vomiting or fever	0	0	2	0	0

## Discussion

In 2004, UNICEF and WHO introduced new guidelines for the management of acute diarrhea in children. The guidelines recommended use of zinc along with ORS because of the results of numerous clinical trials demonstrating that zinc treatment helps to reduce the duration and severity of illness and prevent early recurrence [2,3]. Zinc and ORS are not isolated in the marketplace, however. Caregivers and consumers have access to a range of products for treating diarrhea in children. Some are appropriate, and some are not. In addition, caregivers often use more than one product or intervention during a diarrheal episode. What has been poorly understood is how caregivers perceive zinc relative to other available interventions in areas where caregiver preferences have not been significantly affected by introduction campaigns or pilot projects.

The survey reported in this paper provides indications of how zinc is perceived and the attributes ascribed to it by caregivers in a rural, low–resource setting. About half of the surveyed women reported that their first intervention was some kind of home remedy, which could include homemade sugar/salt solution, foods, liquids, or herbs. In addition, antibiotics were given as the first or second intervention by 46% of caregivers. For about one–half of the respondents, zinc was the third or fourth intervention introduced. This sequence of treatments may reflect access and availability. The ingredients for home remedies are usually readily accessible and do not require a visit to a provider, making it an easy first choice. Antibiotics are more widely available than zinc through private–sector providers, drug vendors, and some shops. During the survey, zinc tablets were generally only available from public sector medical providers, clinics, or hospitals, which tend to be less accessible.

Despite the heavy use of antibiotics, many of the caregivers expressed overwhelmingly positive views of zinc treatment. Eighty–eight percent indicated that zinc was their preferred intervention, and only 5% preferred antibiotics.

Of the 100 zinc users, 75 reported receiving ORS with the zinc. Of these, 59 actually used ORS during the course of the reference illness. Ideally, all 75 who received ORS should use it. However, the fact that some did not may reflect the lack of satisfaction with ORS as a treatment. Only 6% indicated ORS was their preferred treatment choice.

Consistent with previous studies, our survey results indicate that caregivers generally do not follow the recommended 10–day course of zinc treatment, even when they know they should do so [8]. Caregivers typically stop treatment once the child has returned to normal. Two–thirds of respondents reported giving zinc for fewer than 10 days. A large percentage indicated that they knew the correct regimen but for some reason chose to not follow it.

One–third indicated that they gave more than the recommended daily dose of one 20 mg tablet per day. Typically, those who gave more gave two tablets in a day. There may be an attitude of “more is better.” In addition, given that most also gave antibiotics at some point, those who gave more than the daily dose of zinc may be mimicking a dosing regimen more familiar to them, one more similar to the regimen of antibiotics. Programs that promote zinc as an alternative to antibiotics and that seek to position zinc as a medicine may want to monitor dosing behaviors for appropriateness.

Recent studies suggest that 5–day course of zinc may be similar to a 10–day course in its effect on duration of illness [9]. Caregivers may be more likely to comply fully with the shorter course of treatment because it corresponds to the typical duration of illness and may reinforce satisfaction and confidence of self–efficacy. In addition, a 5–tablet pack would undoubtedly make the product much less expensive. More research is needed to assess the efficacy of a shorter course.

Previous studies in controlled settings have indicated that the introduction of zinc has been associated with decreased use of antibiotics [10]. The results of this survey suggest that zinc treatment has a very strong appeal among caregivers who have used it and is perceived as a highly efficacious product to stop diarrhea. More importantly, the vast majority indicated that they preferred zinc over any of the other options available to them, including antibiotics.

The small sample size and the quota sampling methodology need to be considered when evaluating the survey results. The survey was not intended to be representative of all caregivers. Instead, it provided a snapshot of current perceptions. The quota sample was used to allow for simplicity in locating and interviewing a population segment matching a specific criterion outside of a controlled research environment–namely, caregivers who had used zinc to treat diarrhea in the past 6 months. Because of the 6–month time frame, the survey is subject to recall bias. However, we believe the responses are predictive of future behaviors. In addition, knowing that the survey topic was about zinc may have biased the respondents towards a more positive perception of zinc. Finally, in most instances, respondents reported receiving the zinc treatment free of charge, which may have biased the stated preferences. We do not know how costs associated with obtaining the product would influence perceptions and behaviors.

The survey results suggest that zinc has the potential to supplant antibiotics as the diarrhea treatment of choice because of the strong perception that it stops diarrhea. However, treatment regimens for zinc remain unwieldy and unrealistic, perhaps unnecessarily. In addition, the availability of zinc is limited primarily to public–sector providers. Greater access and availability, beyond the public clinic or hospital, may accelerate uptake and sustained use. More research is needed to determine the preferences and perceptions of zinc when cost is a factor.
